# Placental Inflammation and Fetal Injury in a Rare Zika Case Associated With Guillain-Barré Syndrome and Abortion

**DOI:** 10.3389/fmicb.2018.01018

**Published:** 2018-05-16

**Authors:** Kíssila Rabelo, Luiz J. Souza, Natália G. Salomão, Edson R. A. Oliveira, Lynna de Paula Sentinelli, Marcelle S. Lacerda, Pedro B. Saraquino, Fernando C. Rosman, Rodrigo Basílio-de-Oliveira, Jorge J. Carvalho, Marciano V. Paes

**Affiliations:** ^1^Laboratório de Ultraestrutura e Biologia Tecidual, Universidade do Estado do Rio de Janeiro, Rio de Janeiro, Brazil; ^2^Faculdade de Medicina de Campos, Campos dos Goytacazes, Rio de Janeiro, Brazil; ^3^Laboratório Interdisciplinar de Pesquisas Médicas, Instituto Oswaldo Cruz, Rio de Janeiro, Brazil; ^4^Laboratório de Modelagem Molecular, Instituto de Química Orgânica, Universidade Federal do Rio de Janeiro, Rio de Janeiro, Brazil; ^5^Anatomia Patológica, Hospital Municipal Jesus, Rio de Janeiro, Brazil; ^6^Anatomia Patológica, Universidade Federal do Estado do Rio de Janeiro, Rio de Janeiro, Brazil

**Keywords:** Zika virus, immune response, Guillain-Barré syndrome, fetal infection, histopathology

## Abstract

Zika virus (ZIKV) is an emerging virus involved in recent outbreaks in Brazil. The association between the virus and Guillain-Barré syndrome (GBS) or congenital disorders has raised a worldwide concern. In this work, we investigated a rare Zika case, which was associated with GBS and spontaneous retained abortion. Using specific anti-ZIKV staining, the virus was identified in placenta (mainly in Hofbauer cells) and in several fetal tissues, such as brain, lungs, kidneys, skin and liver. Histological analyses of the placenta and fetal organs revealed different types of tissue abnormalities, which included inflammation, hemorrhage, edema and necrosis in placenta, as well as tissue disorganization in the fetus. Increased cellularity (Hofbauer cells and TCD8^+^ lymphocytes), expression of local pro-inflammatory cytokines such as IFN-γ and TNF-α, and other markers, such as RANTES/CCL5 and VEGFR2, supported placental inflammation and dysfunction. The commitment of the maternal-fetal link in association with fetal damage gave rise to a discussion regarding the influence of the maternal immunity toward the fetal development. Findings presented in this work may help understanding the ZIKV immunopathogenesis under the rare contexts of spontaneous abortions in association with GBS.

## Introduction

Zika virus (ZIKV) is an emerging mosquito-borne pathogen that belongs to the *Spondweni* serocomplex of the *Flavivirus* genus, Flaviviridae family (Didier and Gublerb, 2016). Zika fever emerged in Latin America in 2015–2016 and rapidly became a worldwide public health concern (Singh et al., 2016; Slavov et al., 2016). This massive outbreak highlighted possible correlations between the infection and dangerous complications such as Guillain-Barré syndrome (GBS) (Araujo et al., 2016; Malkki, 2016) and congenital microcephaly (Araujo et al., 2016; Garcez et al., 2016). In the absence of specific treatment or vaccine, only in Brazil the number of accumulated infections was estimated between 440,000 and 1,300,000 (Bogoch et al., 2016), with a prevalence of microcephaly of nearly 100 cases per 100,000 live births (Ventura et al., 2016). As an outcome, several unanswered questions, especially regarding the circumstances that may explain a possible connection between the infection and these complications, became a relevant matter of debate.

Initial attempts to model the vertical ZIKV transmission included investigations using immunocompetent mice (Cugola et al., 2016; Vermillion et al., 2017). As these animals are in general resistant to the infection due to virus inability to circumvent the interferon-α/β response (Grant et al., 2016), these models are limited in providing mechanistic explanations to describe pathogenesis. In alternative approaches, genetically modified animals, which are knockout for interferon receptors (IFNARs) or downstream signaling targets, such as IRF3 and IRF7, were employed for investigation (Miner et al., 2016; Yockey et al., 2016). In these reports, despite the characterization of injuries in the fetal brain and the viral escape through the trans-placental route, the animal immunological restriction limited the comprehension of the host response upon infection. In a recent report, an animal model of ZIKV infection involving pregnant non-human primates revealed that, upon prolonged viremia, several fetal tissues, as well as the maternal-fetal interface, were affected (Nguyen et al., 2017). Subjects were found to respond to ZIKV with proliferation of CD16^+^ NK cells and CD8^+^ effector T cells. In addition, the maternal-fetal interface was marked by suppurative placentitis and deciduitis with variable mineralization and necrosis. While reports based on ZIKV-infected non-human primates are valuable due to deep similarities between macaques and human pregnancies, for a better description of the immunopathological events and their impact toward the maternal-fetal link it would be ideal to explore human case samples.

Based on the above considerations, here we investigated a rare Zika case, in which an infected 28-year-old pregnant patient was diagnosed with GBS and had a spontaneous abortion.

## Background

### Clinical presentation

All procedures in this work were approved by the FIOCRUZ Ethics Committee for studies with Zika case and control (CAEE: 65924217.4.0000.5248). The legal representative (mother) of the involved patient provided written consent for the publication of data.

A 28-year-old woman, pregnant, black, housekeeper, from São Francisco do Itabapoana, RJ, was admitted to the hospital on June 29th 2016, presenting weakness in the lower limbs and a consequent inability to ambulate for the previous week. The patient affirmed not having experienced similar episodes previously and claimed to be free of comorbidities. The patient reported rash on her limbs, pruritus and vomiting approximately 1 month before admission to the hospital. Initial obstetric examinations showed globular abdomen, unlimited uterus and absence of vaginal bleeding. The fetal heartbeat was not detected in the Sonar Doppler. Transvaginal ultrasonography evidenced a single fetus with longitudinal status and cephalic circumference suggesting 15 weeks of gestational age, normohydramnios, lack of heartbeat, and non-apparent active movements, which lead to the diagnosis of death and retained fetus (stillbirth). The patient was admitted to curettage and remained in hospital for 30 days. Analysis of the cerebrospinal fluid obtained by lumbar puncture and exams of the patient's peripheral blood revealed normal parameters (Table S1). The IgM serology for Dengue, Chikungunya, Zika, Epstein Barr and Cytomegalovirus were non-reactive (in further sections of this paper, diagnosis of ZIKV infection was confirmed by specific staining in placental and fetal tissues). The patient evolved to ascending and symmetrical flaccid tetraparesis, paresthesia, areflexia, presenting hands with pendular movement, dysautonomia (resting tachycardia and hypertension) and signs of respiratory insufficiency (mild dyspnea at rest which worsens upon effort), characterizing the GBS. The patient was further treated with intravenous immunoglobulin (30 g/day) for five days, atenolol 50 mg 12/12 h, amlodipine 5 mg 24 h, motor rehabilitation, respiratory physiotherapy and psychological intervention. In the second week of disease evolution, the neurological examination showed symmetric flaccid tetraparesis, motor incoordination, global areflexia and sensitive disorders (tactile and thermal distal hypoesthesia). Neurological follow up during and after the hospitalization period are described in Table S2. During the fourth week of disease progress, the patient was admitted to electroneuromyography, which revealed peripheral, acquired, chronic, symmetrical, diffuse, myelin and axonal (predominantly myelin), sensory and motor polyneuropathy: a condition that affected the peripheral nerves of the upper and lower limbs. In addition to that, the patient showed signs of neurogenic myopathy and muscular denervation. Together, these findings were compatible with polyradiculoneuropathy. By the end of the fourth week, the patient was discharged from the hospital under prescription of antihypertensive drugs and still being followed up by motor physical therapy. After 40 days of hospital discharge, the patient was orthostatic, walking-dependent for small distances and with pain sequels in the lower limbs. Five months later, residual sequels were still present with autonomous difficult scarvant gait. After 12 months, the gait patterns were regular; however, the speed and execution of upward and downward movements remained affected. Finally, the patient returned to her daily activities. Methods performed in this work using the placenta and fetal organs are described in Supplementary Material.

## Results

### Investigation of the placental tissue

#### ZIKV infection leads to histopathological damage in placenta

**Figure 1:**
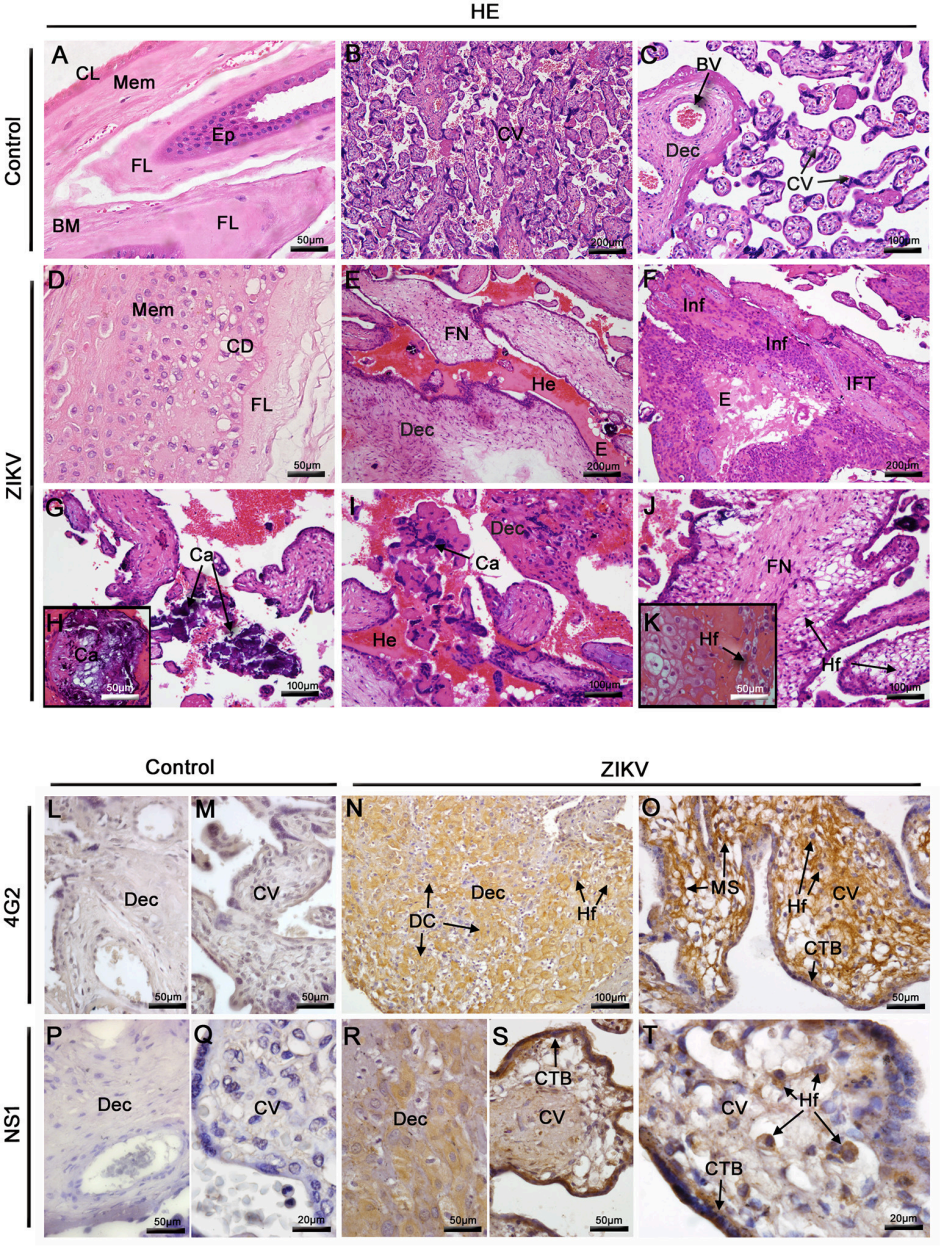
Histopathological analysis of the placenta and detection of ZIKV. **(A–C)** Placenta of a non-ZIKV patient stained with H.E. and presenting normal features: membrane (Mem), (MB) basal membrane, (FL) fibroblastic layer, (CL) compact layer, (Ep) epithelium, chorionic villi (CV), maternal decidua (Dec), and blood vessels (BV). **(D–K)** Sections of ZIKV-infected placental tissue stained with H.E., showing abnormalities in membrane, with cellular degeneration (CD), in the decidua and chorionic villi, including fibrinoid necrosis (FN), hemorrhage (He), mononuclear inflammatory infiltrate (Inf), infarct (IFT), calcification (Ca) and Hofbauer cells with clear cytoplasm (Hf). **(L,M,P,Q)** The flavivirus E protein and NS1 antigens of ZIKV were not detected by immunohistochemistry in the control placenta. **(N–O)** Detection of ZIKV E protein in decidual cells (DC), cytotrophoblasts (CTB), mesenchymal cells (MS) and Hofbauer cells (Hf) of the infected placenta. **(R–T)** The NS1 protein of ZIKV was also detected by immunohistochemistry in decidual cells (DC), cytotrophoblasts (CTB) and Hofbauer cells (Hf).

The histopathological analysis considering the patient's placenta showed relevant damage in the membrane, maternal decidua and chorionic villi. We can highlight large areas of hemorrhage, diffuse fibrinoid necrosis and inflammatory infiltrates formed by mononuclear cells. In addition, regions with cell degeneration and macrophages with clear cytoplasm in membrane and chorionic villi, decidual edema and macrophages were also found. We also noted a decrease in blood vessels of chorionic villi. The decidual region showed dense calcification, which is commonly observed only during the third trimester of gestation (Figures 1D–K). As expected, control samples showed regular arrangement of decidual parenchyma. Controls also exhibited normal chorionic villi, syncytotrophoblasts, cytotrophoblasts and endothelial cells (Figures 1A–C).

The patient's placental tissue was screened for the detection of ZIKV NS1 protein and E protein using immunohistochemistry. Of note, the anti-NS1 antibody used in these assays is ZIKV specific, thus, is able to differentiate ZIKV from other flaviviruses. While the viral antigens were detected in samples from the affected patient, no immunostaining was observed in samples considering the control placenta (Figures 1L,M,P,Q). E structural viral proteins were detected in decidual cells of the maternal portion, cytotrophoblasts and mesenchymal cells of chorionic villi (Figures 1N,O). In the placental portion toward the fetal side, the NS1 protein was detected in cytotrophoblasts, Hofbauer cells of chorionic villi and also in decidual cells (Figures 1R–T). Viral detection occurred mainly within the cytoplasmic region of cells with minor to indistinguishable staining in the nuclear area. This staining pattern strongly suggests that viral replication occurred in these target cells.

#### Characterization of cell subpopulations, colocalization with virus and quantification of cytokine-producing cells

**Figure 2:**
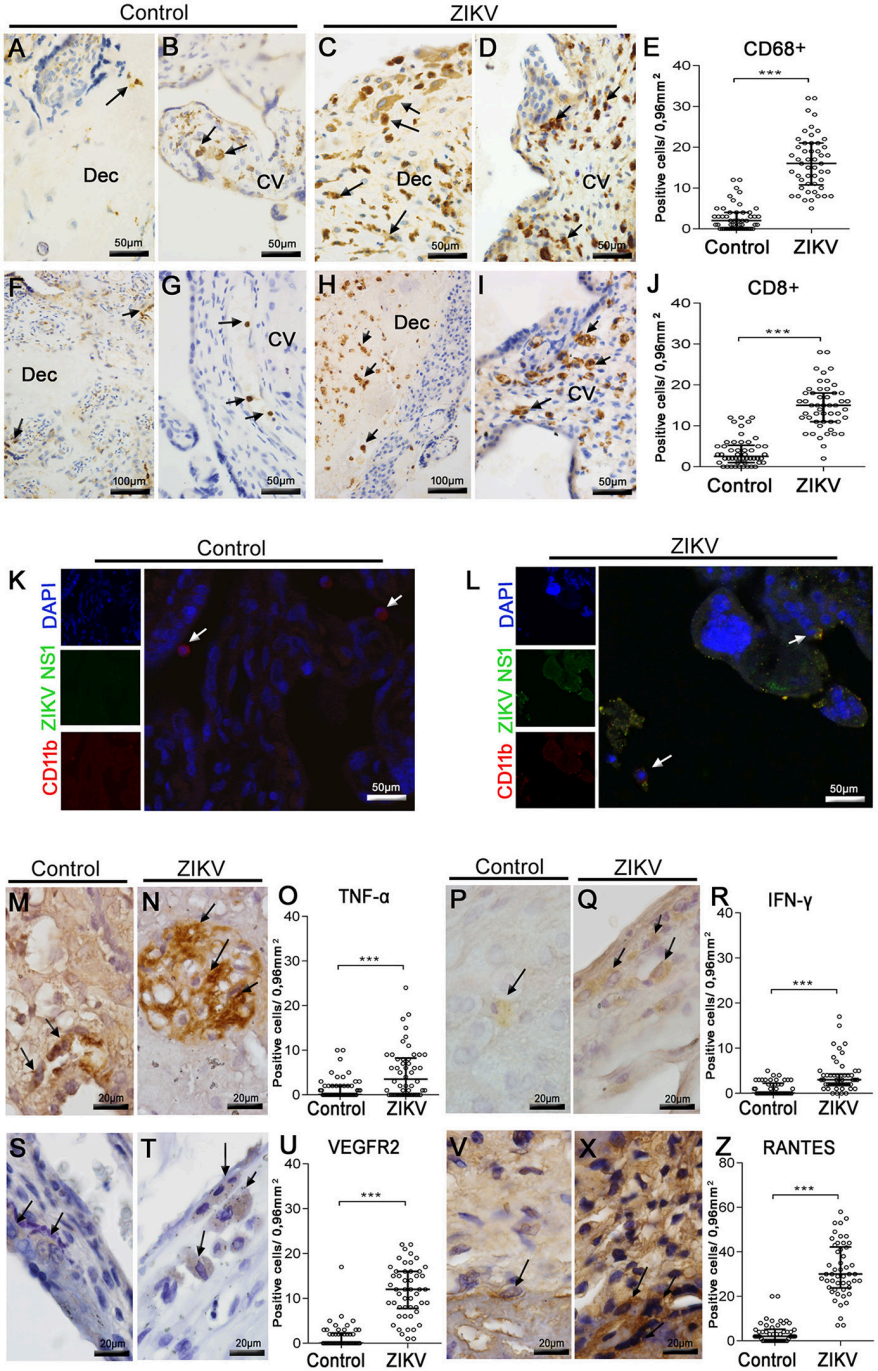
Characterization of mononuclear cell subpopulations in ZIKV-infected placental tissue, colocalization with virus and cytokine-producing cell profile. **(A,B)** Detection of CD68^+^ cells (Hofbauer cells) by immunohistochemistry in decidua and chorionic villi of control placenta, respectively. **(C,D)** Hofbauer cells in decidua and chorionic villi of ZIKV infected placental tissue, respectively. **(F,G)** Detection of CD8^+^ cells by immunohistochemistry in decidua and chorionic villi of control placenta, respectively. **(H,I)** CD8^+^ cells immunostained in decidua and chorionic villi of ZIKV-infected placental tissue, respectively. **(E,J)** Quantification of CD68^+^ and CD8^+^ cells in ZIKV case and control, respectively. **(K,L)** Colocalization by immunofluorescence of the NS1 protein (fluorescent green) and CD11b for identification of leukocytes (fluorescent red). Nuclei were stained using DAPI (fluorescent blue). **(K)** ZIKV NS1 antigen was not detected in the control placenta. **(L)** Cells presenting dual staining (green and red) were observed in the ZIKV-infected placenta. **(M,N)**. Detection of TNF-α in cells of chorionic villi of control and ZIKV infected placenta by immunohistochemistry, respectively. **(P,Q)** Production of IFN-γ in cells of membrane of control and ZIKV infected placenta, respectively. **(S,T)** VEGFR2-expressing cells of decidua in control and ZIKV infected placenta, respectively. **(V,X)** Detection of CCL5/RANTES in cells of chorionic villi of control and ZIKV infected placenta, respectively. **(O,R,U,Z)** Quantification of the number of cells expressing TNF-α, IFN-γ, VEGFR2, and CCL5/RANTES, in ZIKV case and control, respectively. Asterisks indicate differences that are statistically significant between groups (^***^p < 0.001).

Since the histopathological analysis showed inflammatory infiltrates in both maternal and fetal areas, we proceeded with immunohistochemical characterization of the cell types present in this tissue. For this, we used anti-CD68 antibodies to stain the Hofbauer cells and anti-CD8/ anti-CD4 antibodies for phenotype arriving lymphocytes. Staining with anti-CD68 revealed an increase in hyperplasic Hofbauer cells spread in chorionic villi and decidua basalis (Figures 2C,D). While CD8^+^ cells were found in the same areas (Figures 2H,I), CD4^+^ cells were not detected within the tissue. The control tissue showed low density of positive cells (Figures 2A,B,F,G). After quantification considering 50 distinct fields, the numbers of both CD68^+^ and CD8^+^ cells were significantly increased (6- and 4-fold, respectively) in the placenta of the Zika patient, when compared to the control (Figures 2E,J).

Further evidence for ZIKV infection in specific cell subpopulations was provided by immunofluorescence assay. In this case, CD11b^+^ cells (red fluorescence, which we considered as the mononuclear cells) costained with anti-ZIKV NS1 signals (green fluorescence) within several areas of the patient's placenta (Figure 2L). Under this analysis, ZIKV NS1 was also detected in placental cells that were negative for anti-CD11b. As expected, no positive reactions against NS1 were observed in the control tissue (Figure 2K).

To better characterize the inflammatory process in the patient's placenta, we also investigated the local expression of cytokines related to inflammation. Under this approach, we verified the expression of: TNF-α and IFN-γ, due to their well-known participation in a pro-inflammatory context; and VEGFR2 and RANTES/CCL5, since these markers are implicated with altered vascular permeability (Chen et al., 2008; Dalrymple and Mackow, 2012). TNF-α was detected in Hofbauer cells of decidua and chorionic villi (Figure 2N), while expression of IFN-γ was found mostly in macrophages of membrane and decidua (Figure 2Q). The expression of VEGFR2 was found also in macrophages throughout the placental membrane (Figure 2T). The chemokine RANTES/CCL5 was detected mainly in the endothelium and in Hofbauer cells located within the chorionic villi and decidua (Figure 2X). Cells expressing all these cytokines were found in the control tissue, but in smaller amounts (Figures 2M,P,S,V). The numbers of cells expressing all these considered markers were significantly increased in the Zika patient's placenta, when contrasted with the non-Zika control tissues (Figures 2O,R,U,Z).

### Investigation of the fetal tissues

#### Histopathological alterations in fetal organs caused by ZIKV

**Figure 3:**
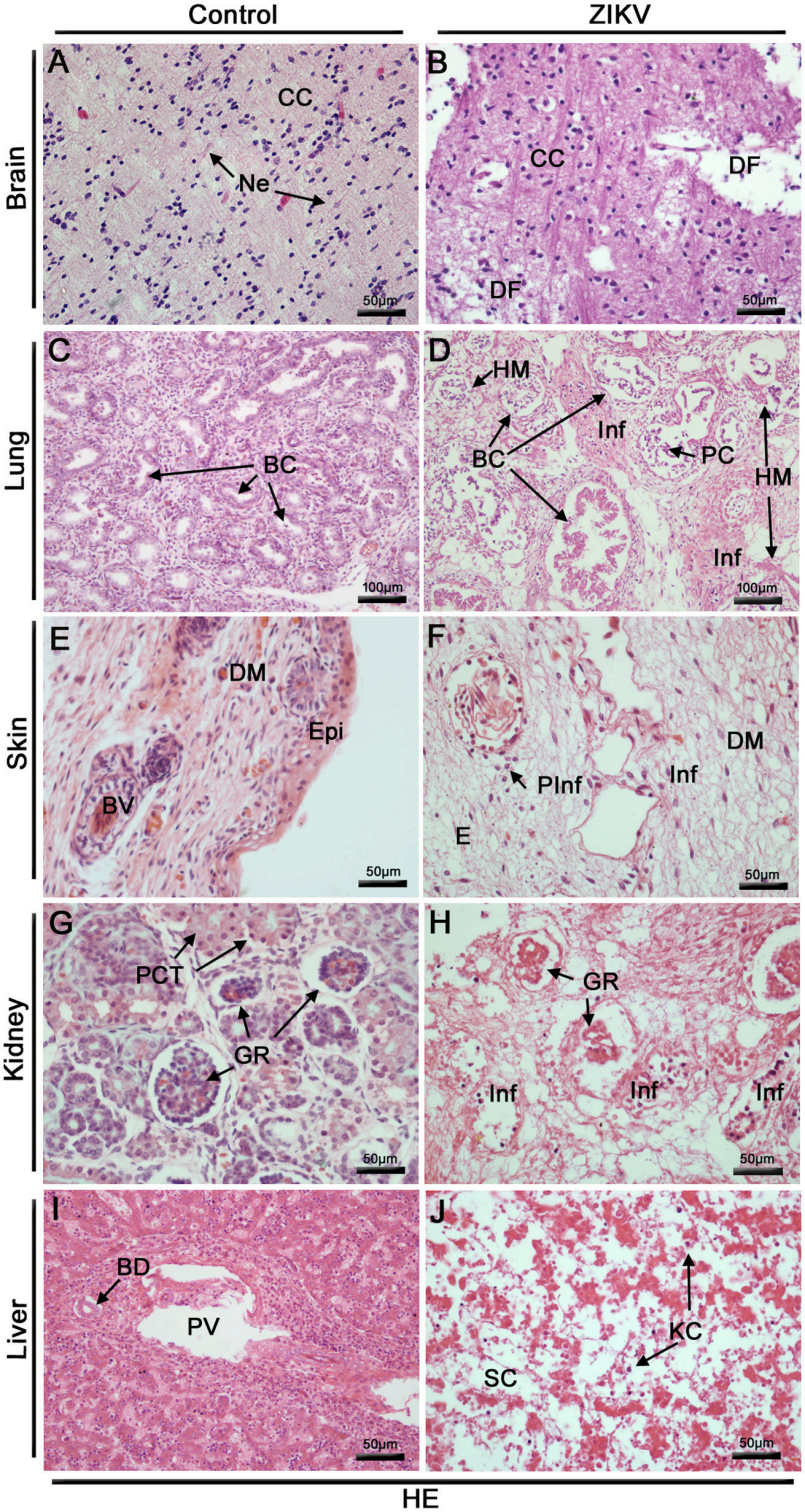
Histopathological analysis of the fetal organs. **(A–J)** All tissues were stained with H. E. **(A)** Brain of a non-ZIKV case presenting normal aspect: cerebral cortex (CC) and neurons (Ne). **(B)** Brain of a ZIKV infected fetus, presenting areas of degenerated nerve fibers (DF). **(C)** Lung section of a control fetus showing normal bronchioles (BC). **(D)** Injuries in fetal lung infected by ZIKV: disorganized bronchioles (DBC) with loss of cylindrical appearance, focal areas of hyaline membrane (HM), diffuse mononuclear infiltrates (Inf) and peeled cells of respiratory epithelium (PC). **(E)** Skin dermis (DM) of a non-ZIKV case presenting normal aspect, epidermis (Epi), blood vessel (BV). **(F)** Skin dermis of a ZIKV infected fetus, with perivascular and mononuclear infiltrate (PInf and Inf) and areas of edema **(E)**. **(G)** Kidney of a non-ZIKV case presenting normal aspect, with normal glomerulus (GR) and proximal contorted tubules (PCT). **(H)** Kidney sections showing injuries, including: disorganized renal glomerulus (GR), tubular disarrangement and inflammatory infiltrate (Inf). **(I)** Section of a control liver, with normal bile duct (BD) and portal vein (PV). **(J)** Liver of a ZIKV infected fetus, presenting dilatation of sinusoidal capillaries (SC), hepatic parenchymal disorganization and Kupffer cells (KC).

The histopathological analysis of the brain tissues collected from the Zika patient's fetus revealed diffuse areas of edema, disorganization of the cerebral cortex layers, mainly in the layer of polymorphic cells and degenerate nerve fibers (Figure 3B). The analysis of the lung tissues revealed several damaged areas with disorganization of the bronchioles architecture associated with focal areas of hyaline membrane. Other alterations in the lungs included regions of septal thickening, increased cellularity, necrosis in the respiratory epithelium accompanied by cell detachments and the presence of mononuclear cell inflammatory infiltrates (Figure 3D). The skin from the affected fetus presented diffuse areas of edema associated with perivascular lymphocytic infiltrate in the dermis region (Figure 3F). In the kidneys, some areas of glomeruli ischemia were observed causing loss of the tubule architecture and its degeneration. This observation was associated with focal mononuclear cell infiltrates (Figure 3H). Liver fetal samples from the Zika patient exhibited severe parenchyma and circulatory disorganization. In this organ, the most prominent lesions were the cellular and matrix degenerations that were associated with increased numbers of Kupffer cells (Figure 3J). As expected, when considering the samples extracted from the control fetus, all the analyzed sites (brain, lungs, skin, kidneys and liver) presented regular structures (Figures 3A,C,E,G,I).

#### Detection of ZIKV antigens in fetal organs

**Figure 4:**
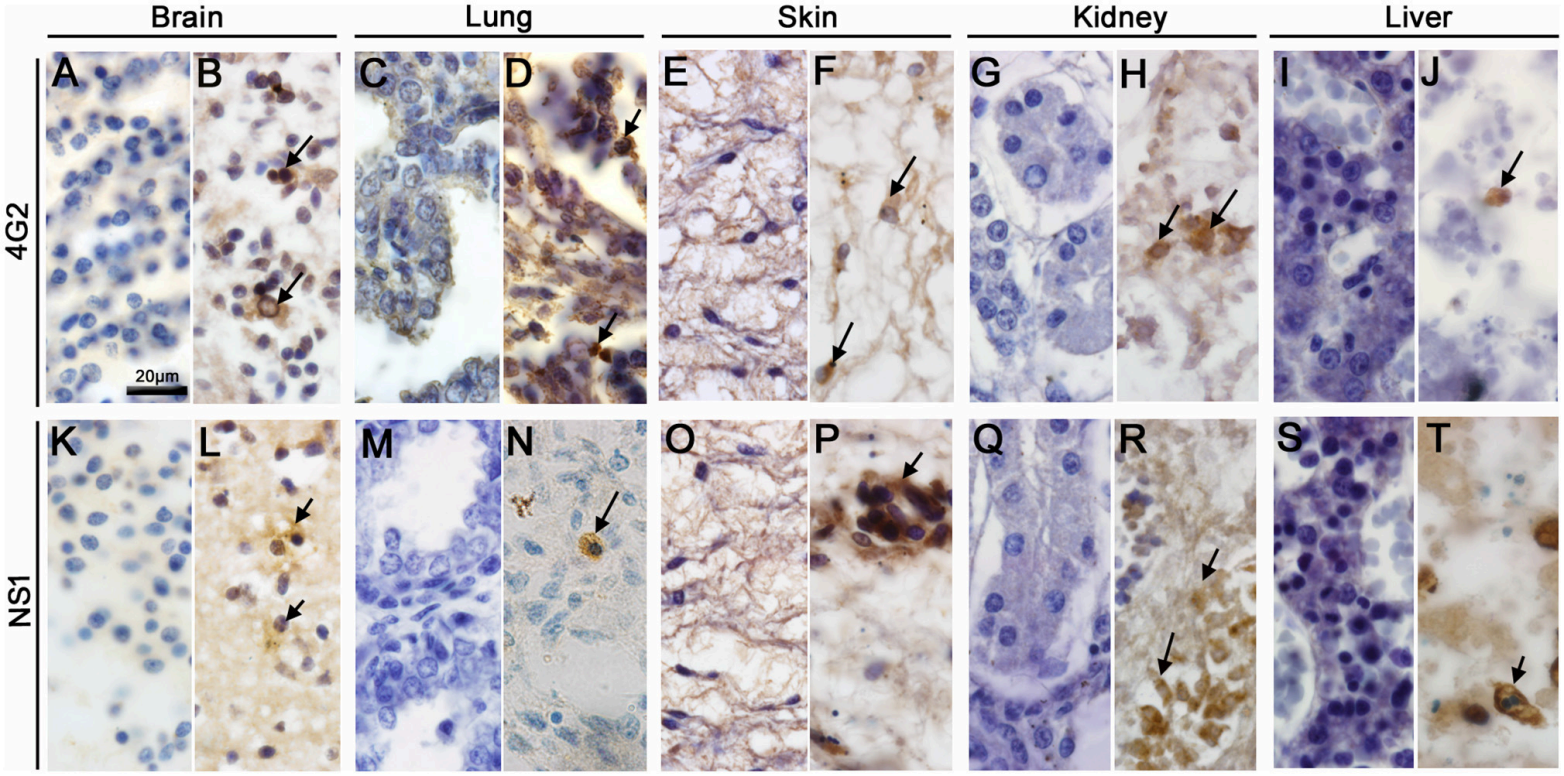
Detection of ZIKV antigens in fetal organs. **(B,D,F,H,J,L,N,P,R,T)** The flaviviral E and ZIKV-NS1 protein were detected in all tissues studied. **(B,L)** Microglial cells and neurons of the brain tissue were positive for ZIKV E protein and NS1, respectively. **(D,N)** ZIKV E and NS1 proteins were detected in alveolar macrophages. **(F,P)** Section of a skin dermis showed mononuclear cells of an inflammatory infiltrate and endothelial cells stained respectively to ZIKV E and NS1 proteins. **(H,R)** ZIKV E protein and NS1 detection in macrophages of the kidney. **(J,T)** Detection of ZIKV E protein and NS1 in hepatocytes, respectively. **(A,C,E,G,I,K,M,O,Q,S)** The E and NS1 antigens of ZIKV were not detected by immunohistochemistry in the control fetus.

Using IHC technique to investigate the Zika patient's fetus we detected the flaviviral-E and ZIKV-NS1 proteins in microglial cells and neurons within the cerebral cortex of the brain tissue (Figures 4B,L). In the lung, these proteins were detected in alveolar macrophages (Figures 4D,N), in mononuclear cells of skin (Figures 4F,P), in macrophages of the kidneys (Figures 4H,R), and, finally, in hepatocytes and Kupffer cells of the liver (Figures 4J,T). No E or NS1 immunostaining was observed in samples from the control organs (Figures 4A,C,E,G,I,K,M,O,Q,S).

## Discussion

The incidence of GBS in Brazil has been evidently increasing after the ZIKV outbreak (Oehler et al., 2014). A recent study showed ZIKV cases with neurological alterations similar to those found in our study, defined as GBS. In these cases, the viremia appeared to persist for longer than normal (Brito Ferreira et al., 2017). While the relationship between Zika fever and GBS still relies solely on epidemiological data, the description of the viral influence toward congenital malformations has become less enigmatic. In this work, we investigated a rare Zika case, which was associated with GBS and spontaneous retained abortion during the 15th week of fetal development. Viral infection was characterized in placental and in several fetal tissues. As found previously (Schaub et al., 2017), this scenario suggested that the case was involved with a high or persistent viremia. Histological analysis of the patient's placenta and fetal organs revealed different types of tissue abnormalities, which included inflammation, hemorrhage, edema and necrosis in placenta and tissue disorganization in the fetus. The patient presented negative IgM serology for several viruses, including ZIKV; however, ZIKV could be detected directly in tissue samples. Based on this, any of the tested viruses (dengue, chikungunya, Epstein-Barr, and Human Citomegalovirus) could also potentially be contributing to the outcome. Nonetheless, given the peculiarity of the clinical presentation, the epidemiological aspect involved, and obviously, the characterization of ZIKV in several areas, we believe that ZIKV may have contributed as the major component for the observed alterations. Of note, the present case happened in an area that had no incidence of yellow fever, which also reduces a probable influence of this disease in the observed results.

In the particular case of the observed placental alterations, one fact that drew our attention was the local increase of numbers of Hofbauer cells in association with the viral infection and pro-inflammatory cytokine production. Infection of Hofbauer cells during the antenatal period not only reflects the critical failure of the protective arrangement, but also highlights a potential pathway for ZIKV vertical transmission. In fact, several research groups have demonstrated either histologically (Noronha et al., 2016; Rosenberg et al., 2017; Rabelo et al., 2017) or by isolated cultures/explants (Jurado et al., 2016; Quicke et al., 2016; Tabata et al., 2016) that these placental macrophages are highly permissive to ZIKV replication. This observation matches the findings from Rosenberg and colleagues, that in a histological study of a Zika case also detected proliferation and hyperplasia of such resident placental cells (Rosenberg et al., 2017). Another indication that ZIKV-infected placental cells were targeted by immune activation was the detection of TCD8^+^ cell infiltrates within the tissue. As broadly investigated previously, TCD8-mediated cellular immunity is apparently critical for host's defense against ZIKV infection (Huang et al., 2017; Ngono et al., 2017; Pardy et al., 2017; Wen et al., 2017). Since we found elevated expression of local pro-inflammatory cytokines (IFN-γ and TNF-α), one hypothesis to explain this scenario is that ZIKV-infected Hofbauer cells may have contributed to the establishment of a chemotactic environment for the arrival of specific lymphocytes.

The considerations exposed above gave rise to a little explored discussion when considering the maternal-fetal link under a viral influence: the maternal immune activation (MIA). Initial thoughts considered pregnancy as a temporary immunosuppressed condition that would be necessary to allow a successful fetal development (Medawar, 1948; Silasi et al., 2015). Nowadays, MIA is thought to be a complex process that changes in a dynamical fashion as the pregnancy evolves (Mor and Cardenas, 2010; Racicot et al., 2014; Silasi et al., 2015). Hypothetically, this entire process culminates in a maternal environment designed to sustain and to protect the pregnancy. In general, when the placenta is targeted by viruses, this organ presents an outstanding capacity to hold back infection, and consequently, to prevent the virus from spreading toward the developing fetus (Romero et al., 2007; Cardenas et al., 2010; Ouyang et al., 2014; Bayer et al., 2015). Conversely, what we saw in the Zika case exposed in this work is much closer to an exception of the above outlook. Due to a yet unknown mechanism, ZIKV seems to hold a unique capacity to circumvent MIA and therefore promote relevant infection and inflammation throughout the placental tissue. Considering the patient's placental conditions, we hypothesize that this fact probably created a bridge between the maternal infection and the effects observed in the developing fetus.

The patient's placental dysfunction caused by ZIKV infection, given the local inflammation and possible altered vascular permeability (as evidenced by the over-expression of RANTES/CCL5 and VEGFR2), may have impaired the normal balance of this hormonal distribution and consequently negatively contributed to fetal development. Other authors have also proposed that placental and decidual inflammation by ZIKV, or other viruses, would critically impact in the normal development of the fetus (Silasi et al., 2015; Mor, 2016). The overexpression of VEGFRs has previously been associated with pathophysiological damage in placentae, while RANTES expression has been found in large quantities in the acute phase of ZIKV infection (Tsatsaris et al., 2003; Tappe et al., 2016). In this sense, a new assumption is proposed for the circumstances by which ZIKV is able to break through the biological placental barrier and to debilitate the pregnancy as a whole.

Although the fetal control tissue samples were at 15 weeks of embryological development and the organs were not yet mature, we observed large histopathological differences between these and tissue samples infected by ZIKV. These injuries are probably due to prolonged viremia in the mother, leading to GBS, fetal involvement and consequently retained abortion. IHC analysis of the patient's fetal brain revealed that this organ was targeted by ZIKV infection, in special the microglial cell types. Several other reports showed that the developing fetal central nervous system (CNS) is highly permissive to ZIKV replication (Kuivanen et al., 2017; Lin et al., 2017; Qian et al., 2017; Rosenfeld et al., 2017). However, despite the well-known tropism of ZIKV to the developing CNS, our findings showed that in the peculiar case of the studied fetus, the infection went beyond the cerebral structure and was found in several peripheral tissues. The commitment of other fetal sites such as the lungs, skin, kidneys and liver supported an idea that the patient was under high or persistent viremia. At the same time, this observation highlights the possibility of novel target tissues when considering an extreme situation, as noted by the clinical features of the studied patient.

## Concluding remarks

This work describes placental and fetal abnormalities found in a rare Zika case involved with GBS and spontaneous abortion. The clinical scenario gave rise to a novel discussion regarding the influence of maternal immunity toward fetal development. Given the unrecognized prevalence of such an uncommon clinical presentation, samples used in this work are valuable for studying the parallel between the infection and the occurrence of GBS and abortion. Findings from this work may add to the current description of ZIKV congenital pathogenesis.

## Author contributions

KR, MP, and JC designed the study; LJS, ML, PS, FR, and LS collected samples and performed clinical exams; KR, NS, and RBO performed all research experiments for placental and fetal evaluation; KR and EO wrote the manuscript; KR, MP, and JC analyzed the experimental results. All authors gave final approval in the manuscript.

### Conflict of interest statement

The authors declare that the research was conducted in the absence of any commercial or financial relationships that could be construed as a potential conflict of interest.
