# Supplementary material for: Placental Inflammation and Fetal Injury in a Rare Zika Case Associated With Guillain-Barré Syndrome and Abortion
Source: Front Microbiol. 2018 May 16;9:1018. doi: 10.3389/fmicb.2018.01018 (PMC5964188; doi:10.3389/fmicb.2018.01018)
Supplement: Supplementary file 1 [file Table_1.DOCX]

**Supplementary material**

**Table S1.** Analysis of the cerebrospinal fluid obtained by lumbar puncture and exams of the patient’s peripheral blood.

|  | **Liquor** | | |  | |
| --- | --- | --- | --- | --- | --- |
| **Test** | **Results** | | | **Reference value** | |
| Cytology | Global: 10 cel/mm^3^ | | | - | |
|  | Specific: 10% PMN/ 90% MNC/ 0% EOS | | | - | |
| Total proteins | 39 mg/dL | | | 10 - 45 mg/dL | |
| LDH | 148 U/L | | | 71- 207 U/L | |
| Glucose | 50 mg/dL | | | 50 - 80 mg/dL | |
| **Peripheral Blood** | | | | | |
|  | | **29/06/2016** | **08/07/2016** | |  |
| Erythrocytes | | 2.52 | 3.51 | | 4.0 – 5.2 millions/ mm^3^ |
| Hemoglobin | | 8.3 | 11.2 | | 12 - 16 g/dL |
| Hematocrit | | 24,1 | 32,4 | | 35 - 47% |
| Leucocites | | 11,000 | 5,500 | | 4,000 – 11,000/mm^3^ |
| Platelets | | 365,000 | 336,000 | | 150.000 – 400,000/ mm^3^ |
| Erythrocyte sedimentation rate | | 60 | 37 | | 0 - 20 mm/h |
| C-reactive protein | | - | 2.05 | | < 0.80 mg/dL |
| Potassium | | 3.6 | 4.2 | | 3.5 - 5.0 mEql/L |
| Sodium | | 142 | 141 | | 135 - 150 mEq/L |
| Urea | | 23 | 14 | | 15 - 45 mg/dL |
| Creatinin | | 0.50 | 0.67 | | 0.4 - 1.4 mg/dL |
| Aspartate aminotransferase | | - | 32 | | 10 - 37 U/L |
| Alanine aminotransferase | | - | 13 | | 10 - 37 U/L |
| Total proteins | | 6.9 | 9.50 | | 6.0 – 8.0 g/dL |
| Albumin | | 4.0 | 3.31 | | 3.5 – 5.5 g/dL |
