# Supplementary material for: Placental Inflammation and Fetal Injury in a Rare Zika Case Associated With Guillain-Barré Syndrome and Abortion
Source: Front Microbiol. 2018 May 16;9:1018. doi: 10.3389/fmicb.2018.01018 (PMC5964188; doi:10.3389/fmicb.2018.01018)
Supplement: Supplementary file 2 [file Table_2.DOCX]

**Supplementary material**

**Table S2.** Evaluation of the degree of upper and lower limbs muscle strength (upper and lower limbs, respectively) during period of hospitalization and follow up after hospitalization.

| **Time of disease evolution (progression)** | **Force quantification** | | | | **Functional Severity Scale**  **Hughes Clinic** |
| --- | --- | --- | --- | --- | --- |
|  | Upper limbs muscle | | Lower limbs muscle | |  |
|  | Proximal | Distal | Proximal | Distal |  |
| 14 days | III | II | II | 0 | 04 |
| 21 days | IV | II | III | 0 | 05 |
| 35 days | IV | III | III | 0 | 04 |
| 46 days | IV | III | III | 0 | 04 |
| 75 days | V | III | IV | II | 03 |
| 123 days | V | IV | V | II | 03 |
| 188 days | V | V | V | IV | 02 |
| 417 days | V | V | V | IV | 02 |
