# Supplementary material for: Placental Inflammation and Fetal Injury in a Rare Zika Case Associated With Guillain-Barré Syndrome and Abortion
Source: Front Microbiol. 2018 May 16;9:1018. doi: 10.3389/fmicb.2018.01018 (PMC5964188; doi:10.3389/fmicb.2018.01018)
Supplement: Supplementary file 3 [file Table_3.doc]

**Supplementary material**

**Material and Methods**

**Ethics and sample collection**

All procedures performed during this work were approved by the Ethics Committee of the Oswaldo Cruz Foundation/FIOCRUZ for studies with Zika case and control (CAEE: 65924217.4.0000.5248). The legal representative (mother) of the involved patient provided written consent and permission for the publication of data and images.

The delivery and specialized care of the patient were performed in Plantadores de Cana Hospital, in Campos dos Goytacazes, Brazil. The placenta and fetus were immediately collected and fixed accordingly to the techniques described below. Consent and permission were obtained from the patient and the institution for comparison purposes (controls) the following samples were considered: placental tissue obtained from a non-Zika case; and a fetus originated from spontaneous abortion at the same stage of development found in the Zika case. The fetus did not present any other infectious disease.

**Histopathological analysis**

Samples from the placentae and fetal organs were fixed in formalin (10%), dehydrated in ethanol, clarified in xylene and blocked in paraffin resin. Tissue sections were cut (4 µm thick), deparaffinized in three baths of xylene and rehydrated with decreasing concentrations of ethanol (100, 90, 80 and 70 %). Sections were stained with hematoxylin and eosin for 2 min, for histological examination. Stained specimens were visualized by light microscopy (Olympus BX 53F, Japan) and digital images obtained by Image Pro Plus software (Version 4.5). All analyses were performed without prior knowledge of the nature of the samples (blind test).

**Immunohistochemical procedure**

For immunohistochemical studies, the paraffin-embedded tissues were cut (4 µm thick), deparaffinized in xylene and rehydrated with alcohol. Antigen retrieval was performed by heating the tissue in the presence of citrate buffer. Next, tissues were blocked for endogenous peroxidase with 3% hydrogen peroxidase in methanol and rinsed in Tris-HCl (pH 7.4). To reduce non-specific binding, sections were incubated in Protein Blocker solution (Spring Bioscience, USA) for 5 min at room temperature. Placental and fetal samples were then incubated overnight at 4 ºC with anti-human monoclonal antibodies that recognize flavivirus E protein (4G2 - produced in house as described in (Henchal et al., 1982) ), Zika NS1 (Arigo, USA), diluted 1:200. This step was also performed to placental tissue with CD8 (DAKOCytomation, USA), CD68 (Biocare Medical, USA), RANTES/CCL5 (Santa Cruz Biotechnology, USA), TNF-*α* (Abbiotec, USA), IFNγ (Abbiotec), VEGFR2 (Spring Bioscience, USA), all diluted 1:200. In the next day, sections were incubated with a rabbit anti-mouse IgG-HRP conjugate (Spring Bioscience) for 40 min at room temperature. For negative controls, samples were incubated with both antibodies or only with the secondary HRP conjugated antibody. Reactions were revealed with diaminobenzidine (Dako, USA) as chromogen and the sections were counterstained in Meyer’s hematoxylin (Dako).

**Quantification of positive cells by immunohistochemistry**

Slides were evaluated using an Olympus BX 53F microscope. For each specific antibody, 50 images (fields) were randomly acquired at 1000x magnification using the software Image Pro version 4.5 from placentae (zika infected and control). After collecting the frames, positive cells were quantified in each of the 50 fields in every organ and the median of positive cell number was determined. All analyzes were accomplished in a blind test without prior knowledge of the studied groups. After quantification, frames exhibited in figures were selected as to be more informative.

**Immunofluorescence assay and co-staining of NS1 protein/ phenotypic cell markers:**

The paraffin-embedded tissues were cut (4 µm thick), deparaffinized in xylene and rehydrated with decreasing alcohol series. Antigen retrieval was performed by heating the tissue in presence of citrate buffer. In sequence, tissues were blocked with 1% bovine serum albumin (BSA) for 30 minutes and permeabilized with 0.5% Triton X-100 at room temperature. Samples were incubated overnight at 4 ºC with anti-human monoclonal antibodies that recognize Zika NS1 protein (Arigo, USA) and anti-CD11b (Abcam, UK) diluted 1:200 for co-localization. In the next day, sections were incubated with Alexa 488 rabbit anti-mouse IgG (Thermo Scientific) to bind anti-NS1 antibodies and Alexa 555 mouse anti-rabbit IgG (Thermo Scientific, USA) to bind anti-CD11b antibodies. Slides were analyzed under a confocal microscope (Zeiss LSM 510 Meta, Germany).

**Statistical analyses**

Data were analyzed with GraphPad prism software v 6.0 (La Jolla, USA) using Mann-Whitney non-parametric statistical tests. Significant differences between groups were determined considering ****p <* 0.001.

**References**

Henchal, E., Gentry, M., McCown, J., and Brandt, W. (1982). engue Virus-Specific and Flavivirus Group Determinants Identified with Monoclonal Antibodies by Indirect Immunofluorescence. Am J Trop Med Hyg. 31, 830–6.
